# Studies on the metabolism and degradation of vancomycin in simulated *in vitro* and aquatic environment by UHPLC-Triple-TOF-MS/MS

**DOI:** 10.1038/s41598-018-33826-9

**Published:** 2018-10-19

**Authors:** Mengsi Cao, Yanru Feng, Yan Zhang, Weijun Kang, Kaoqi Lian, Lianfeng Ai

**Affiliations:** 1 Department of sanitary inspection, School of Public Health, Hebei Medical University, Shijiazhuang, 050017 China; 2 Hebei Food Safety Key Laboratory, Hebei Food Inspection and Research Institute, Shijiazhuang, 050091 China; 3 Hebei Province Key Laboratory of Environment and Human Health, Shijiazhuang, 050017 China; 4 Hebei Entry-Exit Inspection and Quarantine Bureau, Shijiazhuang, 050051 China

## Abstract

Vancomycin is one of the most commonly used glycopeptide antiobiotics, and as such is an important emerging environmental contaminant. Pharmaceuticals and personal care products (PPCPs), such as antibiotics, are problematic since wastewater treatment processes are not completely effective at removing these chemical compounds. Since wastewater treatment processes are not completely effective, vancomycin occurs in surface water. Vancomycin and its metabolites *in vivo* and degradation products in aquatic environment may lead to undesirable ecological effects that threaten the environment or cause undesirable reactions that affect human health. We aimed to study vancomycin metabolism *in vitro* and its natural degradation in aquatic environment, as well as explore for related metabolites and degradation products. Accordingly, we established four systems, using a constant temperature oscillator at 37 °C for 10 days for vancomycin in activated rat liver microsomes (experimental system), inactivated rat liver microsomes (control system), phosphate buffer saline (PBS system) and pure water (pure water system), as well as an additional system of activated rat liver microsomes without vancomycin (blank system). The metabolism and degradation of vancomycin were studied using a high resolution and high sensitivity ultra-high performance liquid chromatography (UHPLC)-Triple-time of flight (TOF)-mass spectrometry (MS) method in positive ion mode. The compared result of activated rat liver microsomes system and inactivated rat liver microsomes system confirms that vancomycin is not metabolized in the liver. Vancomycin was degraded in the four non-blank incubation systems. The MetabolitePilot 2.0 software was used for screening the probable degradation products, as well as for establishing its associated degradation pathways. Eventually, four degradation products were identified and their chemical structures were deduced. The results of this study provide a foundation for evaluation of the effects of vancomycin and its degradation products on environmental safety and human health in the future.

## Introduction

Pharmaceuticals and personal care products (PPCPs) are widely concerning as a group of emerging environmental contaminants due to their possible threats to aquatic environment and human health^1,2^. PPCPs comprises numerous diverse products, including pharmaceuticals (human clinical medicine and veterinary drugs) and active ingredients in personal care products (PCPs)^3^. The deposited PPCPs may either retain their original concentrations and structures or be metabolized and converted into other active (or inactive) compounds during their lifespan in aquatic matrices^4^. Indeed, not only PPCPs itself, but also their metabolites and degradation products in the environment can have a potential negative impact on the environment, living organisms and human health by means of migration or accumulation^5,6^.

As a commonly used clinical drug, vancomycin is one of the PPCPs that is of significant concern. Among all the glycopeptide antibiotics in clinical use, vancomycin is the most commonly used to treat serious infections like endocarditis, pneumonia and meningitis, caused by vancomycin-susceptible Gram-positive organisms, such as methicillin-resistant *Staphylococcus aureus* (MRSA)^7,8^. In Italy and Germany, treated urban discharges can be an entry route of vancomycin into surface water, because wastewater treatment processes are not completely effective in removing vancomycin^9^. In French Rivers, vancomycin has been detected at concentrations reaching 90 ng/L^10^. Vancomycin is ingested by humans and animals through drinking water and may be biotransformed into related metabolites *in vivo*. Vancomycin may be degraded in an aquatic environment to produce related degradation products. Vancomycin and its metabolites and degradation products in aquatic environment may lead to detrimental ecological effects that threaten the environment or produce adverse reactions that affect human health. The ecological risk of antibiotics in the aquatic environment is a growing concern. Vancomycin, as an antibiotic, may have acute or chronic ecotoxicity on bacteria, algae, invertebrates, and fish in the aquatic environment^9^. The increased risk of renal toxicity due to vancomycin was found to be likely associated with its higher daily dose and longer duration of ingestion by eating, drinking and treating^11,12^. Drug toxicity may result from the parent compound or from its metabolites^13–15^. Nevertheless, no data on the metabolism or degradation of vancomycin are currently available. Therefore, we aim to find related metabolites or degradation products by studying vancomycin metabolism *in vitro* and natural degradation in aquatic environment. This would provide a foundation for the study of the effects of vancomycin and its degradation products on environmental safety and human health in the future.

Drug metabolism research can be divided into *in vivo* and *in vitro* metabolism studies^16–18^. The liver is the main organ for biotransformation, with cytochrome P450 enzyme system playing an important role in drug metabolism^19,20^. As drug metabolism may be altered by NADPH concentrations *in vivo* and *in vitro*^21^, appropriate concentrations of NADPH should be used. The *in vitro* metabolism model based on liver microsomes has been widely used in drug metabolism research and has the advantages of being fast and accurate, and having a high throughput^22–24^. *In vitro*, liver microsomal incubation simulates the physiological environment of metabolism in the liver.

To the best of our knowledge, there are only a few studies about the metabolism of vancomycin. Aboleneen *et al*., used high-performance liquid chromatography (HPLC) to determine the serum concentration of vancomycin and the major and minor isomers of its degradation product, CDP-1, in patients with renal impairment^25^. White *et al*., used a microbiological assay, HPLC and a polarisation fluoroimmunoassay to measure the vancomycin concentration in phosphate-buffered saline, peritoneal dialysis effluent fluid and human serum after daily sampling for 10 days of incubation at 37 °C, but vancomycin metabolites were not determined^26^. The aquatic ecological environment is complex and diverse, containing organics and mineral salt, etc^27,28^. Accordingly, the existing rat liver microsomes system and phosphate buffer saline (PBS) system may simulate the aquatic environment containing organics and mineral salt respectively. Besides being a simulation of the basic aquatic environment, the pure water system is also a contrasting environment to other types of aquatic environments. Therefore, we elected to establish five systems including activated rat liver microsomes (experimental system), inactivated rat liver microsomes (control system), activated rat liver microsomes without vancomycin (blank system), phosphate buffer saline (PBS system) and pure water (pure water system) to study vancomycin metabolism *in vitro* and natural degradation in an aquatic environment.

For drug metabolism or degradation studies, liquid chromatography mass spectrometry (LC-MS) has become the most commonly used technique to discover previously unreported metabolites and degradation products. This is especially true for time-of-flight (TOF) mass spectrometry (MS) instruments, which have the advantages of excellent sensitivity, high resolution, accurate mass detection, fast analysis, and comprehensive detection of the nature of the metabolite^29,30^. Widespread use of the AB SCIEX triple TOF 5600 MS/MS system is attributed to its online data acquisition, which is followed by comprehensive data processing techniques including extracted ion chromatography (EIC), mass defect filtering (MDF), product ion filtering (PIF), neutral loss filtering (NLF) and isotope pattern filtering. The information dependent acquisition (IDA) criteria are provided to the ions that match the mass defect window to obtain the MS/MS spectra. This enables the chemical structure of the compound to be deduced^31^. Thus, in this study, samples were analyzed by ultra-high performance liquid chromatography connected to an AB SCIEX triple TOF 5600 MS/MS system to identify related metabolites and degradation products. This study has an important hygienic significance for evaluation of the effects of vancomycin on environmental safety and human health in the future.

## Materials and Methods

### Chemicals and reagents

Vancomycin hydrochloride was purchased from Dr. Ehrenstorfer GmbH (Augsburg,Germany). Nicotinamide adenine dinucleotide phosphate (NADPH) and phosphate buffer saline (PBS) were purchased from Shanghai Yisheng Biotechnology Co., Ltd (Shanghai, China) and Sangon Biotech Co., Ltd (Shanghai, China), respectively. Analytical grade magnesium chloride (MgCl_2_) was obtained from Yongda Chemical Reagent Co., Ltd (Tianjin, China). Acetonitrile and formic acid of HPLC grade were purchased from Thermo Fisher Scientific Co., Ltd (Shanghai, China) and DikmaPure Technologies Company (Beijing, China), respectively. HPLC grade methanol was purchased from Sunrise Chem, INC (New York, USA). Pure water was prepared by the Milli-Q system made in Millipore Corporation (Billerica, USA).

### Standard solutions preparation

Vancomycin and NADPH were dissolved in pure water at the exact concentrations of 6 g/L and 10 mmol/L when they were used, respectively. MgCl_2_ was previously dissolved in PBS at a concentration of 13.3 mmol/L.

### Chromatographic and mass spectrometric conditions

UHPLC-Triple-TOF-MS/MS analysis was performed on a Shimadzu LC-30A UHPLC system (Kyoto, Japan) consisting of an autosampler (SIL-30AC), a binary pump (LC-30AD) and a column oven (CTO-30A) connected to an AB SCIEX triple TOF 5600 MS/MS system (Redwood, USA) equipped with Duo-Spray ion source. The compound separation was conducted with a Poroshell 120 EC-C18 (2.1 × 150 mm, 2.7 μm, Agilent InfinityLab, USA) chromatographic column maintained at 40 °C. The mobile phases A (0.1% aqueous formic acid) and B (acetonitrile) were used to optimized the gradient elution program, which was operated at follows: 0–1 min, 5% B; 1–9 min, 5–10% B; 9–14 min, 10–45% B; 14–20 min, 45–90% B; 20–25 min, 90% B for washing the column; 25–25.1 min, 90–5% B; 25.1–30 min, 5% B for equilibrating the column. The constant flow rate was 0.4 mL/min, and the injection volume was 5 µL.

The full-scan MS spectra and MS/MS spectra data were obtained in positive electrospray ionization (ESI) mode by Analyst TF 1.6 software (AB Sciex, USA). The optimized parameters of the high resolution mass spectrometer are shown in Table 1. Full scan mode was applied, the parent ions scan ranged from m/z 100 to 2000 Da with a 200 ms accumulation time and the product ions scan range from 100 to 1500 Da with a 70 ms accumulation time. Simultaneously, the calibration delivery system calibrated mass numbers by every five samples to acquire exact mass of ions. The running time of data independent acquisition (DIA) was 20 min.

**Table 1:** The optimized parameters of the high resolution mass spectrometer.

Parameters	Value
Ion source temperature (°C)	550
IonSpray voltage floating (V)	5500
Ion source gas 1 (psi)	50
Ion source gas 2 (psi)	50
Curtain gas (psi)	35
Collision energy (eV)	46
Declustering potential (eV)	80

### Preparation of rat liver microsomes

Ten male Sprague-Dawley (SD) rats (250 ± 20 g) were provided by the Experimental Animal Research Center at Hebei Medical University. The animal experimentation were approved by the Ethics Committee of Hebei Medical University (Approval Number 2018012). Rat liver was removed immediately after decapitation and then weighed blotting the excess blood with a filter paper. Liver microsomes were prepared by differential centrifugation after the liver was repeatedly washed with a cold solution (10 mmol/L Tris-HCl, 250 mmol/L sucrose and 1 mmol/L ethylene diamine tetraacetic acid (EDTA) at pH 7.4), shredded and homogenized on ice. A full description of the procedures can be obtained elsewhere^32^. Eventually, the Lowery protein assay was used to measure protein concentrations in the rat liver microsome suspension^33^.

### Experimental design

In this study, five systems (experimental, control, blank, PBS and pure water systems) were established. The five systems each included 11 samples, in a final volume of 200 µL. All systems, except the blank system, contained a final vancomycin concentration of 60 mg/L^26^. The experimental, control and blank systems contained PBS and MgCl_2_ at a final concentration of 3.3 mmol/L. The experimental and blank systems contained 1 mg/mL of activated rat liver microsome protein, and the control system contained 1 mg/ml of inactivated rat liver microsome protein. After preheating the incubation system at 37 °C for 5 min, NADPH at 1 mmol/L was added to the experimental and blank systems, while the same volume of pure water was added to the control system. Vancomycin was diluted in PBS and pure water in the PBS system and pure water system, respectively (details in Table 2). One sample from each system was placed directly in the freezer at −20 °C (0 h), and the remaining samples of the incubation systems were placed in a constant temperature oscillator at 37 °C for 1–10 days. One sample from each system was removed every 24 hours for ten consecutive days, adding 1 mL pre-cooled methanol immediatel yafter removal to the experimental, control and blank systems. All samples were stored at −20 °C until further analysis.

**Table 2:** Five systems of vancomycin.

200 µL System	MgCl_2_ (µL)	NADPH (µL)	Active rat liver microsomes(µL)	Inactiverat liver microsomes(µL)	Vancomycin (µL)	PBS (µL)	Pure water (µL)
experimental	50	20	20	—	2	108	—
control	50	—	—	20	2	108	20
blank	50	20	20	—	—	108	2
PBS	—	—	—	—	2	198	—
pure water	—	—	—	—	2	—	198

### Pretreatment of samples

All the samples were centrifuged (10,625 g for 10 min) including experimental, control and blank systems. The supernatant was completely transferred to a clean glass tube and dried with a slow stream of nitrogen at 37 °C. The dried substances were dissolved with 200 µL 30% methanol before determining.

### Quality control (QC) sample

A QC sample of vancomycin in pure water was prepared with a concentration of 60 mg/L. The same QC samples were injected at the start of the run and then again every eleven samples.

### Data analysis

Comparing of the raw data in five systems by MetabolitePilot 2.0 software (AB Sciex, USA), we can acquired the information on probable metabolites or degradation products, such as biotransformation, molecular formula, retention time (RT), m/z values, relative error (ER) and score. According to the full-scan MS spectra and MS/MS spectra of the probable metabolites or degradation products provided by PeakView 2.2 software (AB Sciex, USA) and the structure characterization of vancomycin analysed by Mass Frontier 5.0 software (Thermo Fisher Scientific, USA), the probable chemical structures of the metabolites or degradation products could be calculated.

## Results and Discussion

### Degradation of vancomycin

**Figure 1:**
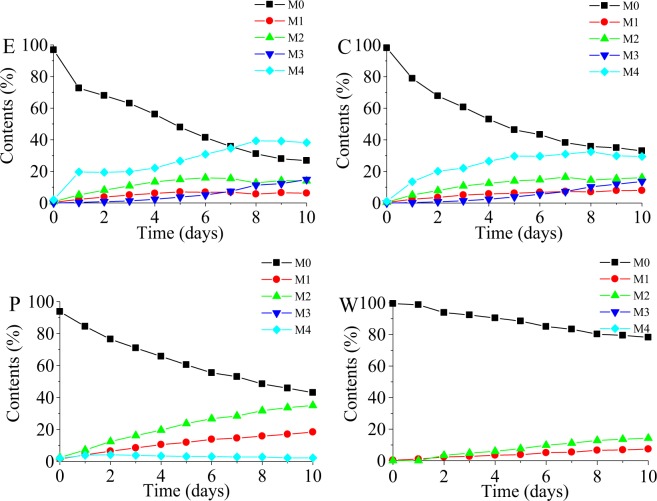
The degradation of vancomycin and the generated trends of four degradation products in experimental system (**E**), control system (**C**), PBS system (**P**) and pure water system (**W**) at 37 °C for 10 days.

During the 1–10-day incubation period, the concentration of vancomycin (M0) decreased in the experimental, control, PBS and pure water systems (Fig. 1). A similar degradation rate in the experimental and control systems over the early time period of the incubation, when the microsomes are viable, may be attributed to the weak effect of activated rat liver microsomes cytochrome P450 enzymes on vancomycin. The results confirmed that vancomycin is not metabolized in the liver and most of the vancomycin ingested in the body is excreted in the urine. The most marked loss of vancomycin was in rat liver microsomes, which was degraded 50% over approximately 6 days. The time to 50% loss of vancomycin in PBS or in water was approximately 9 days and over 10 days, respectively, which showed that vancomycin can be degraded in an aquatic environment. Organics and mineral salt may be favorable to the degradation of vancomycin.

### Mass spectrometry fragmentation patterns of vancomycin

**Figure 2:**
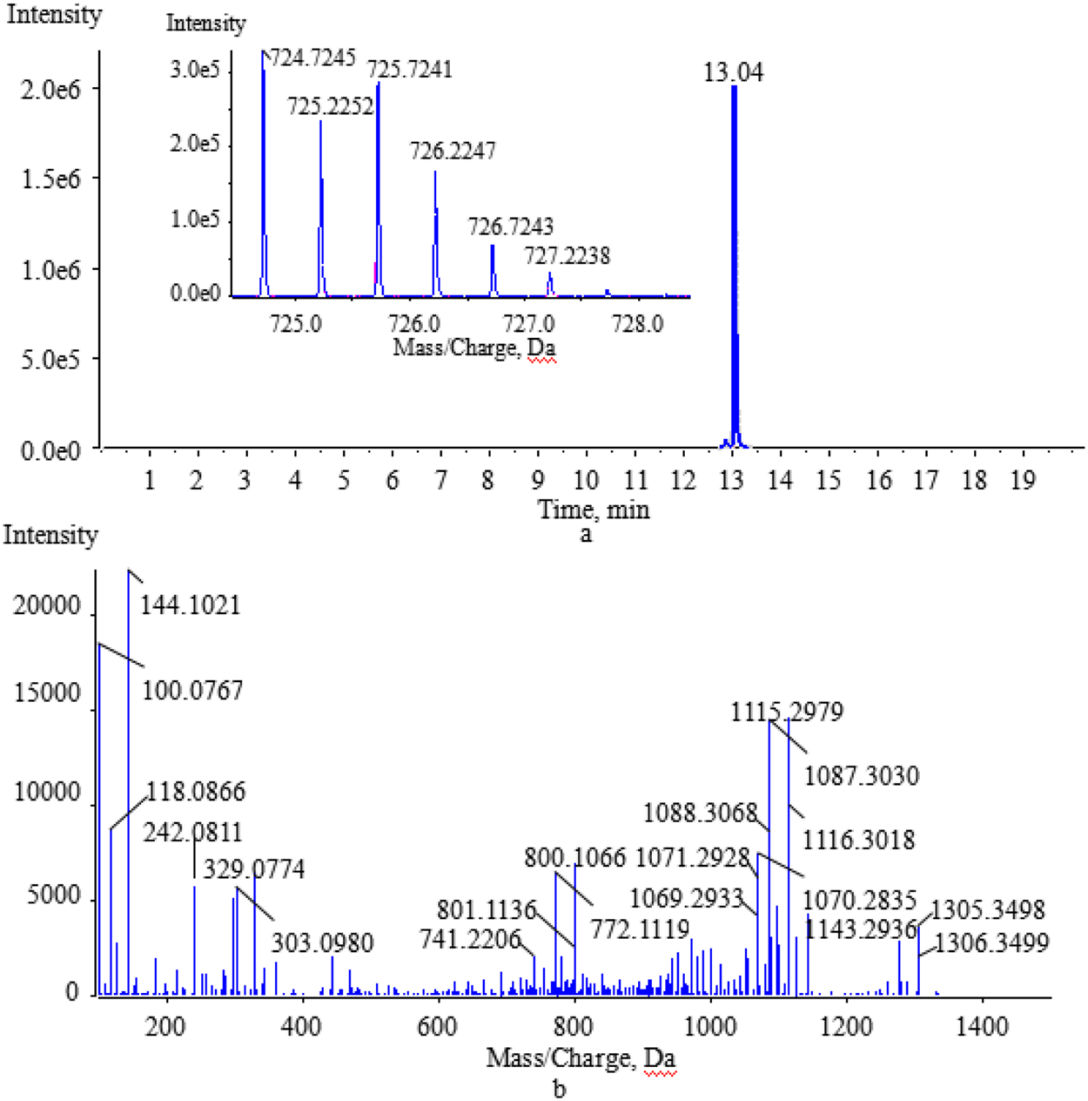
The extract ion chromatogram, MS(**a**) and MS/MS(**b**) spectra of vancomycin.

**Figure 3:**
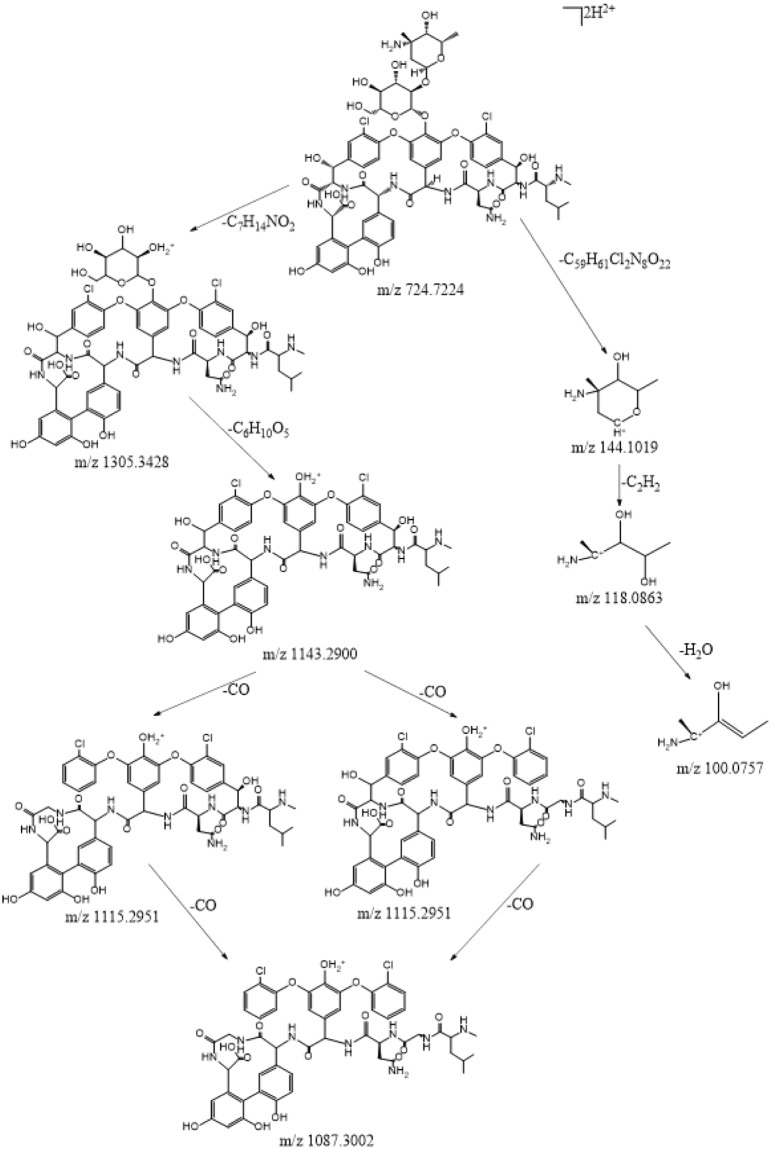
The proposed fragmentation pathways of vancomycin.

The extracted ion chromatogram and the MS fragmentation patterns of vancomycin, reveal that vancomycin eluted at 13.04 min with a parent ion at m/z 724.7246 with two charges (Fig. 2a). The chemical structure of vancomycin is very complex, and includes aminoglycoside and polypeptide. Combined with the MS/MS spectra of vancomycin (Fig. 2b), the MS/MS fragmentation behaviors were analyzed by inference and the ‘Fragmentation Library function’ in the Mass Frontier 5.0 software. The results of the analysis suggested that vancomycin was initially fragmented into the fragment ions m/z 144.1019 (F1) and 1305.3428 (F2). The two fragment ions then produced a series of characteristic product ions at m/z 118.0863 [F1-(-C_2_H_2_)]^+^ (F3), 100.0757 [F3-(-H_2_O)]^+^ (F4), 1143.2900 [F2-(-C_6_H_10_O_5_)]^+^ (F5), 1115.2951 [F5-(-CO)]^+^ (F6,F7) and 1087.3002 [F6,F7-(-CO)]^+^ (F8). The main ions were at m/z 100.0767, 144.1021, 1115.2979, 1087.3030 in the MS/MS spectrum (Fig. 2b)^34–36^. The proposed fragmentation pathways for vancomycin are shown in Fig. 3. The fragment ions at m/z 100.0767, 118.0866 and 144.1021 were produced from fragmentation of the aminoglycoside of vancomycin. Fragmentation of the polypeptide occurred with the loss of two CO to produce characteristic product ions at m/z 1115.2979 and 1087.3030. According to the characteristic fragmentation patterns of vancomycin, the probable chemical structures of the degradation products could be further deduced^37^.

### Identification of the degradation products

#### The procedure to identify likely degradation products

The Triple TOF MS/MS instrument, data independent acquisition (DIA) and various techniques to process the raw data were used to identify likely degradation products.

**Figure 4:**
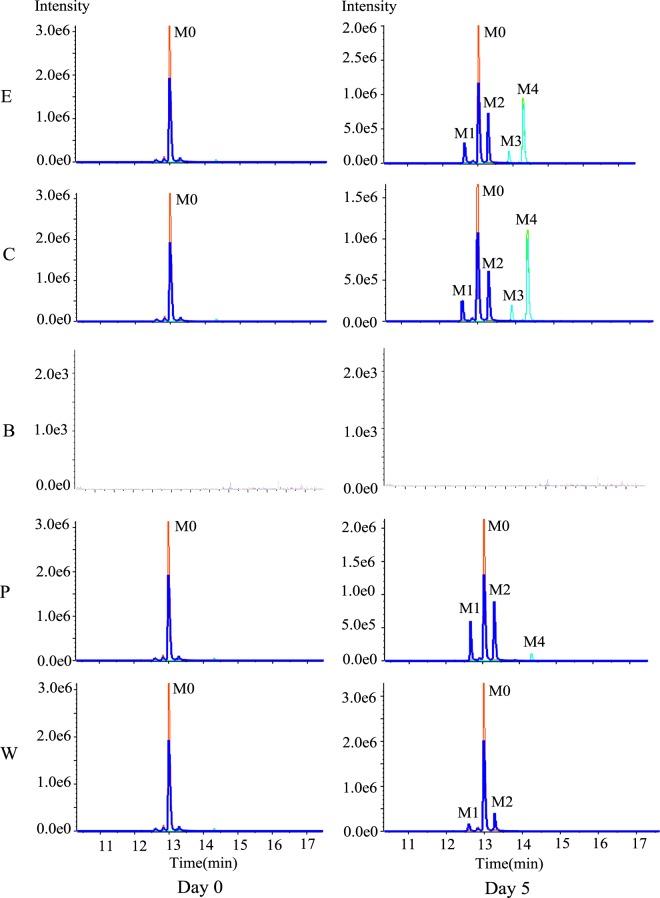
The XIC of vancomycin and the four vancomycin degradation products. M0, M1, M2, M3, M4 in experimental system (**E**) and control system (**C**); no metabolites in blank system (**B**); M0, M1, M2, M4 in PBS system (**P**); M0, M1, M2 in pure water system (**W**).

First, the raw data from the experimental, control, PBS and pure water systems were compared with the blank system and a prepared standard solution of vancomycin using the MetabolitePilot 2.0 software, and the likely degradation products were acquired. Subsequently, the data for likely degradation products were acquired and confirmed using many data-mining functions in the PeakView software, such as extracted ion chromatogram (XIC), information dependent acquisition (IDA) explorer, mass calculator and formula finder. Ultimately, four likely degradation products (M1, M2, M3 and M4) of vancomycin (M0) were identified. The biotransformation pathway, molecular formula, retention time, theoretical and observed mass, mass error and score for vancomycin and each of its four likely degradation products are outlined in Table 3. For high quality and accurate mass spectra, the relative error (RE) between the theoretical and observed values for the ion mass were less than 5 ppm^38,39^. Accordingly, the inferred element composition of the four likely degradation products was acceptable, as the RE for vancomycin and the four likely degradation products was less than 5 ppm. Using the XIC function in the PeakView software, the extracted ion chromatograms of the experimental, control, blank, PBS and pure water systems were obtained by applying the characteristic ion at m/z 724.7245, 725.2171, 725.2167, 731.2164, 730.7239 of vancomycin and the four likely degradation products. The comparison between day 0 and day 5 of incubation is shown in Fig. 4. The chromatograms reveal that M1, M2, M3 and M4 were present in the experimental and control systems. Additionally, no degradation products were detected in the blank system, but M1, M2 and M4 were detected in the PBS system, and only M1 and M2 were detected in the pure water system.

**Table 3:** The information of vancomycin and four degradation products.

Peak ID	Metabolite discription	Molecular Formula	R.T. (min)	Theoretical Mass	Observed Mass	RE (ppm)	MS m/z [M + 2H]^2+^	MS/MS Fragments	Score (%)
M0	Parent	C_66_H_75_Cl_2_N_9_O_24_	13.04	1447.4302	1447.4344	2.9	724.7245	100.0767	97.7
								118.0866	
								144.1021	
								1087.303	
								1115.2979	
								1043.2936	
								1305.3498	
M1	Oxidative Deamination to Alcohol	C_66_H_74_Cl_2_N_8_O_25_	12.64	1448.4142	1448.4197	3.8	725.2171	118.0859	86.6
								144.1014	
								1116.2826	
M2	Loss of CH_3_N + Oxidation and Methylation	C_66_H_74_Cl_2_N_8_O_25_	13.31	1448.4142	1448.4189	3.2	725.2167	100.0759	82.4
								118.0857	
								144.1015	
								1144.2798	
M3	Loss of CH_3_N + N-Acetylation	C_67_H_74_Cl_2_N_8_O_25_	13.9	1460.4142	1460.4185	2.9	731.2164	100.0763	83.8
								144.1022	
								1128.2832	
M4	Loss of water + Oxidation and Methylation	C_67_H_75_Cl_2_N_9_O_24_	14.3	1459.4302	1459.4344	2.9	730.7239	100.076	82.9
								144.1015	
								1099.2981

#### The contents of the degradation products

The relative standard deviation (RSD) of the vancomycin intensity in the QC samples was 14.41%, which was less than 15%, thus indicating that the repeatability and stability were acceptable^40,41^. Based on the definition that the total content of vancomycin and its four degradation products is 100%, the related content of vancomycin and its four degradation products were calculated using the normalization method. The trends for vancomycin and the four degradation products during the 1–10-day incubation period in the experimental, control, PBS and pure water systems are shown in Fig. 1. In the experimental and control systems, M1 and M2 increased with time over the first seven days and then decreased slightly from day 8. Compared with the other three systems, M1 and M2 had the fastest increase in the PBS system, with M2 increasing faster than M1. This may be due to the stimulation and inhibition of the formation of M1 and M2 by the mineral salt and organics, respectively. M3 was only produced in the experimental and control systems. Compared with the other three degradation products, M3 was the lowest produced in the first seven days and then exceeded M1 after seven days. M4 showed an obvious upward trend with the longer time in both the experimental and control systems, and increased at the fastest rate of the four degradation products. M4 showed a trend to increase in the first two days in the PBS system and then decreased slightly from day 3. The M4 content was the lowest in the PBS system, and was not found in the pure water system. A possible explanation for this is that organics were favorable to M3 and M4 production.

#### Structural deduction of the degradation products

**Figure 5:**
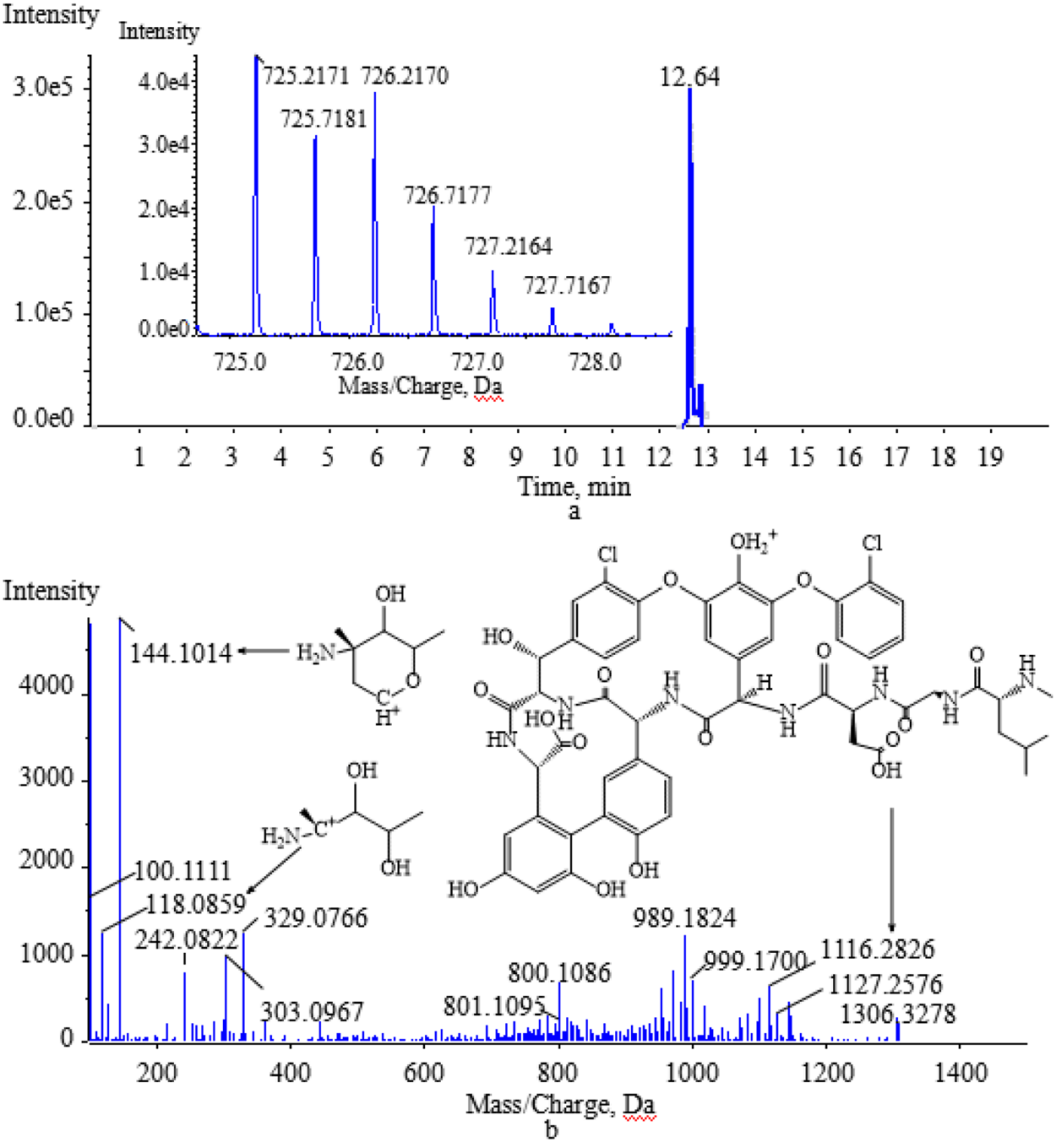
The extract ion chromatogram, MS(**a**) and MS/MS(**b**) spectra of M1.

**Figure 6:**
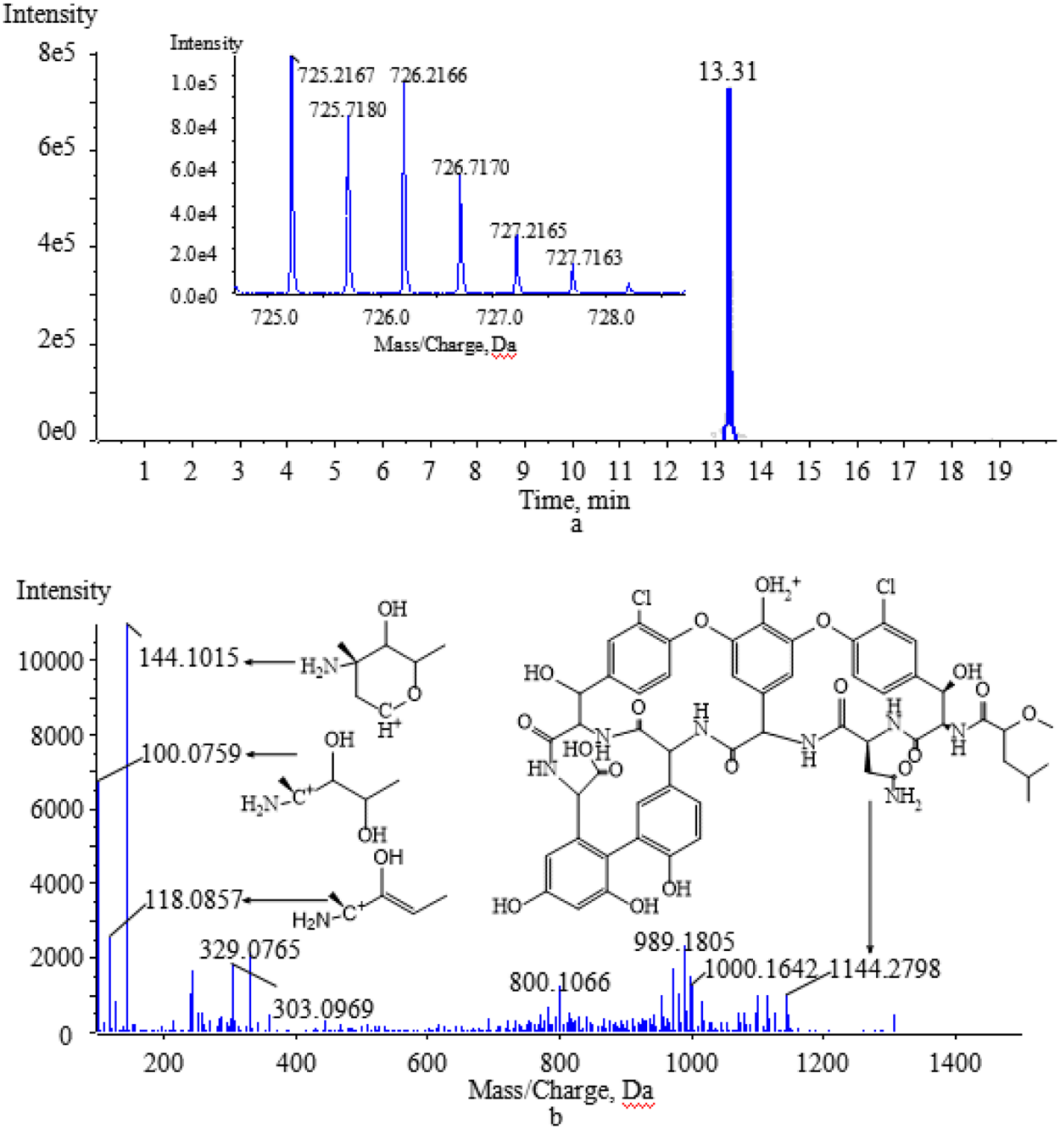
The extract ion chromatogram, MS(**a**) and MS/MS(**b**) spectra of M2.

**Figure 7:**
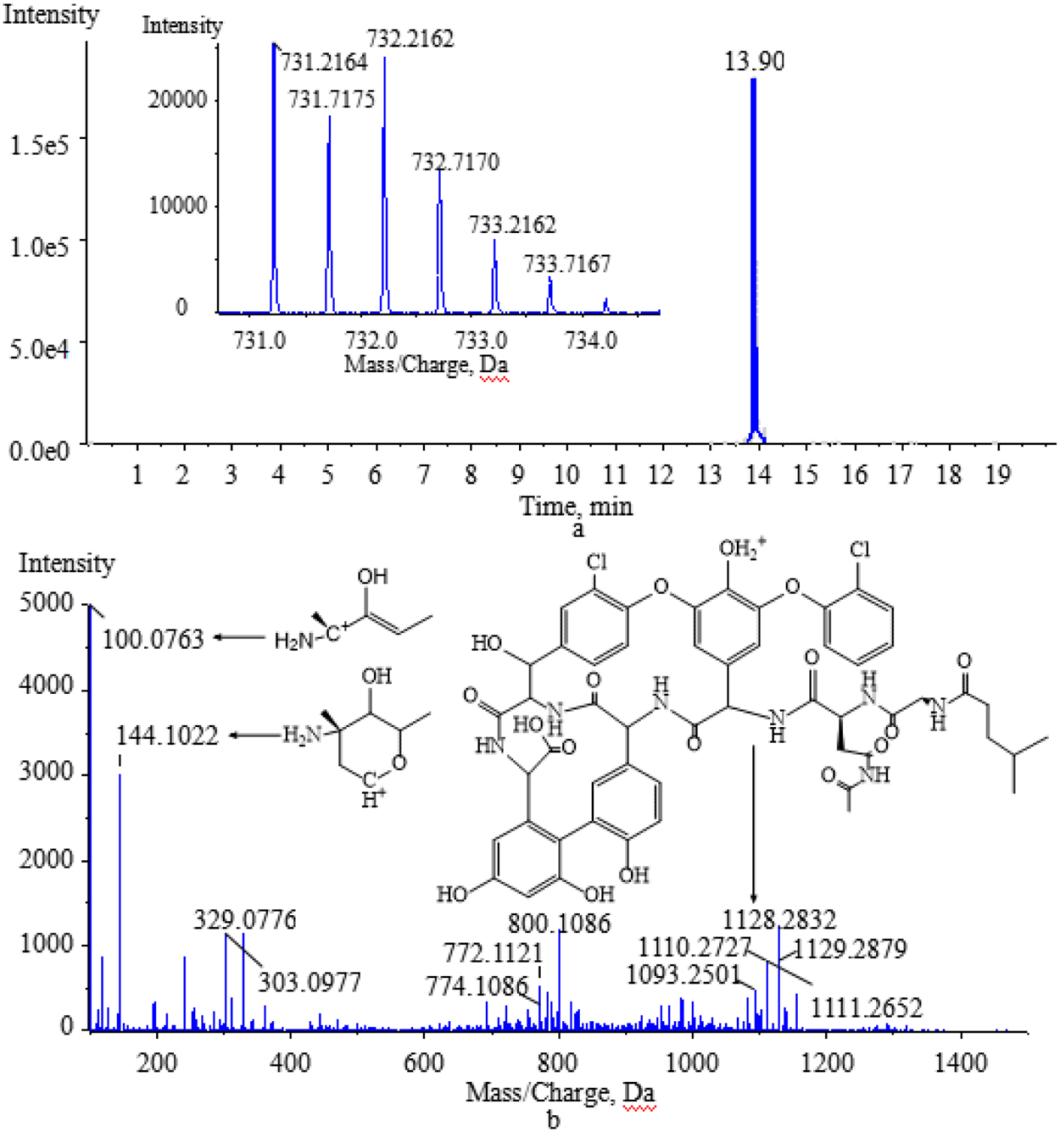
The extract ion chromatogram, MS(**a**) and MS/MS(**b**) spectra of M3.

**Figure 8:**
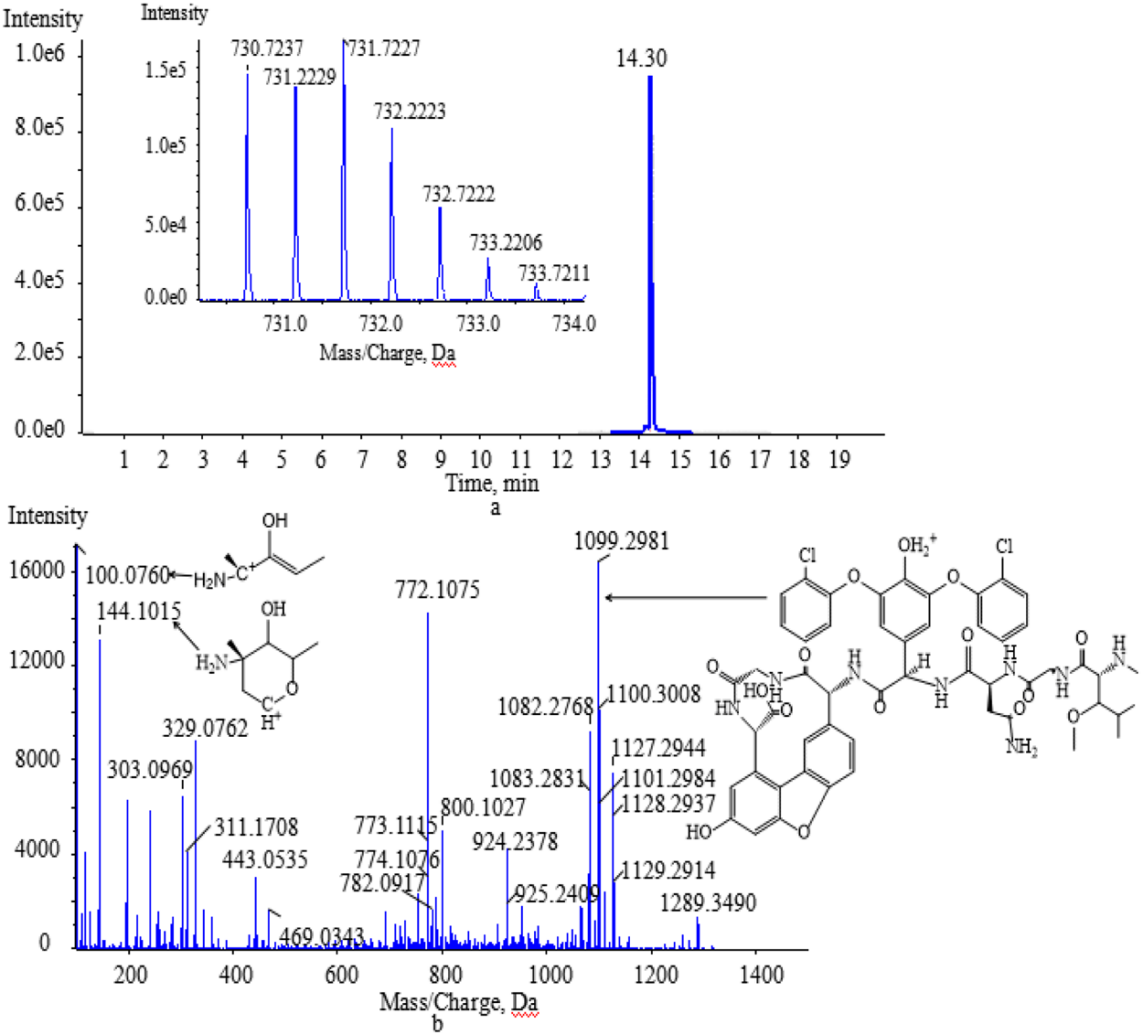
The extract ion chromatogram, MS(**a**) and MS/MS(**b**) spectra of M4.

The degradation products formed by biotransformation generally retain the basic skeleton and some substructures of the original parent molecule. This means that the degradation products may have similar cleavage laws to the raw drugs in HPLC-MS/MS analysis. Vancomycin contains two amino groups (one on the aminoglycoside moiety and the other on the polypeptide component). The MS/MS spectra of vancomycin and its four degradation products (Figs 2b, 5b, 6b, 7b and 8b) reveal that all the compounds contained the same fragment ions at m/z 100.07, 118.08 and 144.10. These fragment ions were produced from the fragmentation of the aminoglycoside fragmentation of vancomycin, thus the amino moiety in the aminoglycoside structure is consistent across all four degradation products. Based on the finding that vancomycin easily loses the CO part from the polypeptide under collision energy, we reasoned that the degradation products had similar principles and this provided the basis for deducing their chemical structures.

The retention time for M1 ([M + 2H]^2+^ at m/z 725.2171) was 12.64 min (Fig. 5a). M1 was acquired by the biotransformation process of oxidative deamination to alcohol, which suggested that M1 was obtained by changing the amino group in the polypeptide structure of vancomycin. M1 lost part of the aminoglycoside and one CO to produce the characteristic product ion at m/z 1116.2826. The likely structure of the characteristic product ion at m/z 1116.2826 is shown in Fig. 5b.

M2 eluted at 13.31 min and had the same protonated ion ([M + 2H]^2+^ at m/z 725.2167) as M1 (Fig. 6a). The biotransformation process for M2 involved the loss of CH_3_N, and oxidation and methylation. Vancomycin contains only one CH_3_N group within the polypeptide structure. M2 lost the part of aminoglycoside to produce the characteristic product ion at m/z 1144.2798 (Fig. 6b). M1 and M2 are isomers, and the polarity of M1 is greater than that of M2 according to the value of the CLogP. As a result, M1 eluted before M2.

Vancomycin lost a CH_3_N group and was N-acetylated to produce M3 ([M + 2H]^2+^ at m/z 731.2164), which eluted at 13.90 min (Fig. 7a). The loss of CH_3_N was the same as for M2, and the position of the change in the amino moiety was the same as for M1. The likely characteristic product ion at m/z 1128.2832 was acquired by M3, which lost the aminoglycoside component and a CO (Fig. 7b).

M4 ([M + 2H]^2+^ at m/z 730.7237) was obtained by the biotransformation process involving the loss of water and oxidation and methylation and eluted at 14.30 min (Fig. 8a). Vancomycin contains many hydroxyl groups, and two adjacent hydroxyl groups could be dehydrated to form ethers. The likely characteristic product ion at m/z 1099.2981 was acquired by M4, which lost the aminoglycoside and two CO moieties (Fig. 8b).

**Figure 9:**
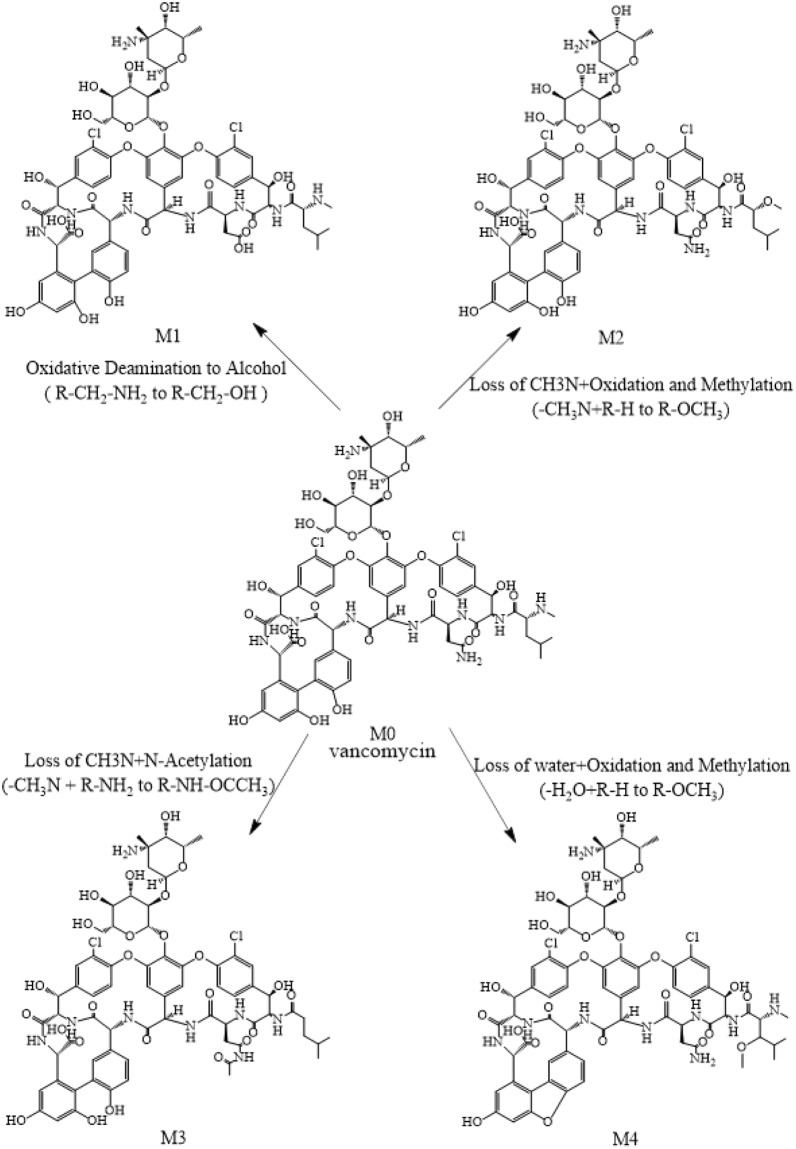
Probable degradation pathways of vancomycin *in vitro* and deductive chemical structure of four degradation products.

The Fragmentation Library Function in the Mass Frontier 5.0 software played a guiding role in the process to deduce the chemical structure of the four degradation products. The chemical structures of the four degradation products are illustrated in Fig. 9. The theoretical and observed MS and MS/MS mass of the four degradation products are listed in Table 4. All relative errors of the ion mass were less than 10 ppm.

**Table 4:** The theoretical and observed MS and MS/MS mass of four probable degradation products of vancomycin.

Peak ID	Theoretical MS m/z [M + 2H]^2+^	Observed MS m/z [M+2H]^2+^	RE (ppm)	Theoretical MS/MS Fragments	Observed MS/MS Fragments	RE (ppm)
M0	724.7224	724.7245	2.9	100.0757	100.0767	10.0
				118.0863	118.0866	2.5
				144.1019	144.1021	1.4
				1087.3002	1087.303	2.6
				1115.2951	1115.2979	2.5
				1043.29	1043.2936	3.4
				1305.3428	1305.3498	5.4
M1	725.2144	725.2171	3.7	118.0863	118.0859	−3.4
				144.1019	144.1014	3.5
				1116.2791	1116.2826	3.1
M2	725.2144	725.2167	3.2	100.0757	100.0759	2.0
				118.0863	118.0857	−5.1
				144.1019	144.1015	−2.8
				1144.274	1144.2798	5.1
M3	731.2144	731.2164	2.7	100.0757	100.0763	6.0
				144.1019	144.1022	2.1
				1128.2791	1128.2832	3.6
M4	730.7224	730.7239	2.0	100.0757	100.076	3.0
				144.1019	144.1015	−2.8
				1099.3002	1099.2981	1.9

#### The degradation pathway of vancomycin

In this study, a total of 4 metabolites were identified. The plausible degradation pathways of vancomycin are shown in Fig. 9. Additionally, the principle of the degradation of vancomycin into four metabolites was described in detail in the “*Structural deduction of the degradation products*” section above. The results showed that the amino group, methylamino group, adjacent hydroxyl group and hydrogens on the alkyl group in the polypeptide were the main degradation sites.

## Conclusions

We used a high resolution and high sensitivity UHPLC-Triple-TOF-MS/MS method in positive ion mode to investigate the degradation of vancomycin in activated and inactivated rat liver microsomes, PBS and pure water systems. The compared result for activated rat liver microsomes system and inactivated rat liver microsomes system confirmed that the effect of activated rat liver microsomes cytochrome P450 enzymes on vancomycin is so weak that vancomycin is essentially not metabolized in the liver. A comparison of the degradation trends of vancomycin in the four systems, reveals that organics and mineral salt may be favorable to the degradation of vancomycin in aquatic environment. Ultimately, four likely novel degradation products were identified. The structural characteristics and cleavage of the four novel degradation products were deduced based on the mass spectra analysis of vancomycin. These finding could be a foundation for future research on the effects of vancomycin and its degradation products on environmental safety and human health.
